# Effect of BMI-stratified management based on the meridian-lymph axis theory on lower extremity lymphedema after gynecological cancer surgery: a randomized controlled trial

**DOI:** 10.3389/fonc.2026.1900880

**Published:** 2026-09-24

**Authors:** Yulin Geng, Hong Zhang

**Affiliations:** Gynecological Oncology, Tongji Hospital, Tongji Medical College of Huazhong University of Science and Technology (HUST), Wuhan, Hubei, China

**Keywords:** gynecological cancer, integrated therapy, lymphedema, meridian theory, obesity

## Abstract

**Background:**

Lower-extremity lymphedema (LEL) is a chronic complication after gynecological cancer surgery. Obesity may aggravate lymphatic dysfunction and reduce the effectiveness of standard physical management. We evaluated a BMI-stratified integrated intervention informed by a meridian-lymph conceptual framework.

**Methods:**

A total of 60 patients with gynecological cancer-related LEL were randomly assigned in a 1:1 ratio to an intervention group (n=30) or a control group (n=30). The intervention group received BMI-stratified meridian massage and dietary and health guidance for 6 months, whereas the control group received compression stockings, health education, and aerobic exercise. Patients were divided into three subgroups by BMI: <24 kg/m², 24–27.9 kg/m², and ≥28 kg/m². The primary outcome was change in affected-limb volume, and quality of life was assessed using LYMQOL. Outcome assessors were blinded to group allocation.

**Results:**

At 6 months, affected-limb volume decreased by 32.1 ± 6.2% in the intervention group and 14.3 ± 5.8% in the control group (P<0.01). LYMQOL scores improved by 15.2 ± 3.1 and 6.8 ± 2.9 points, respectively (P<0.01). Exploratory subgroup analysis suggested a greater volume reduction among participants with BMI ≥28 kg/m² than among those with BMI 24-27.9 kg/m² (P = 0.02). Three participants in the intervention group developed transient skin redness, and no serious adverse events were reported.

**Conclusion:**

In patients with LEL after gynecological cancer surgery, the BMI-stratified integrated intervention was associated with greater improvement in limb volume and quality of life than conventional care over 6 months.

**Clinical trial registration:**

ClinicalTrials.gov, identifier NCT07374835.

## 1 Introduction

Lower extremity lymphedema (LEL) is a common chronic complication after lymph-node dissection or radiotherapy for cervical and endometrial cancer and can substantially impair function, body image, and quality of life (1). Elevated BMI is a recognized risk factor for lymphatic dysfunction. Experimental studies have shown that diet-induced obesity is associated with adipose expansion, reduced collecting-lymphatic contraction frequency and responsiveness to mechanical stimulation, perilymphatic inflammation, increased lymphatic leakiness, and impaired lymphatic transport. Aerobic exercise can partially restore lymphatic pumping and transport even in the absence of weight loss (2, 3). Existing interventions have obvious limitations. Air pressure devices have insufficient tissue penetration in obese patients and may cause discomfort in emaciated patients; multi-layer bandaging easily loosens in high-temperature environments and induces contact dermatitis; kinesiology tape is nearly ineffective in overweight patients due to insufficient adhesion (4–6). These dilemmas highlight the urgent need for BMI-stratified, individualized treatment strategies.

The “meridian-lymph axis” theory provides a potential conceptual perspective for LEL management. Anatomical studies have characterized the superficial lymphatic drainage pathways of the lower extremities, while experimental evidence indicates that neuropeptides such as calcitonin gene-related peptide may modulate lymphatic vessel function (7, 8). However, these findings do not establish a direct anatomical correspondence between specific acupoints and lymphatic vessels, and direct evidence for acupoint-specific regulation of human lymphatic function remains limited. Therefore, in the present study, the “meridian-lymph axis” is used as a clinical conceptual framework rather than as an established anatomical or mechanistic pathway.

Beyond intrinsic lymphatic contractility, extrinsic mechanical forces also contribute to lymph propulsion. Human scintigraphic studies have shown that dynamic and isometric skeletal-muscle contractions increase lymph clearance, supporting a role for muscle contraction and tissue deformation in lymph transport. Experimental studies further indicate that collecting lymphatic vessels are mechanosensitive and can entrain their contractions to oscillatory external pressure, providing a physiological basis for compression-based physical management (9, 10). Together with evidence of obesity-related lymphatic dysfunction (2, 3), these findings provide the established physiological background for the BMI-stratified management strategy evaluated in this study. This paper proposes a meridian-lymph integrated treatment strategy based on BMI stratification. For patients with different BMI levels, manual acupoint pressure was applied at SP6, SP9, SP10, and ST36, with the duration and supportive management adjusted according to BMI stratification. This strategy aims to provide an individualized approach for the management of lower extremity lymphedema (11, 12). This program aims to break through the bottleneck of current physical therapy through anatomical-functional dual targeting, providing a new paradigm for precise LEL management.

## 2 Methods

### 2.1 Study design

This was a single-center, open-label, parallel-group randomized controlled trial. Eligible participants were assigned in a 1:1 ratio to the BMI-stratified integrated intervention or conventional care and followed for 6 months. Because participants and treating staff could not be blinded to the behavioral and manual intervention, outcome assessments were performed by researchers blinded to group allocation. The study was conducted by a multidisciplinary team comprising head nurses, certified lymphedema therapists, and oncologists.

The random allocation sequence was generated before participant enrollment by an independent researcher using a computer-generated random-number list with a 1:1 allocation ratio. The allocation sequence was concealed from the investigators responsible for participant recruitment and baseline assessment. Group assignments were placed in sequentially numbered, opaque, sealed envelopes prepared according to the randomization sequence. After a participant had completed enrollment and baseline assessment, the next envelope in numerical order was opened to determine group assignment. Outcome assessors remained blinded to group allocation throughout the study.

#### 2.1.1 Study subjects and recruitment

Inclusion criteria were age 18–75 years, gynecological cancer treated with lymph-node dissection, and clinically diagnosed postoperative lower-extremity lymphedema according to the criteria of the International Society of Lymphology (ISL). Diagnosis was based on clinical examination, including persistent limb swelling, circumferential limb measurement, and exclusion of edema caused by systemic diseases such as cardiac or renal disorders. Lymphedema severity was classified according to the ISL staging system BMI was categorized as <24, 24-27.9, or ≥28 kg/m².

Exclusion criteria were pre-existing lymphedema, edema primarily attributable to renal or cardiac disease, and severe cognitive or psychiatric disorders that precluded participation.

Sample size was estimated using G*Power 3.1 based on the study protocol. With a two-sided α of 0.05, a statistical power of 80%, and an expected large effect size (Cohen’s d=0.8), the planned sample size was 30 participants per group, resulting in a total sample size of 60 participants.

#### 2.1.2 Follow-up and outcome framework

Outcome assessments were conducted at baseline and 6 months. The primary outcome was the percentage change in affected-limb volume at 6 months. The secondary outcome was the change in LYMQOL score. Adverse events were recorded throughout the intervention period.

The eligibility criteria, intervention procedures, primary and secondary outcomes, and assessment time points were consistent with the registered protocol (ClinicalTrials.gov identifier: NCT07374835). During the study implementation, additional prespecified secondary outcomes, including GCLQ, L-Dex, and tissue hardness, were not assessed due to practical limitations and were therefore excluded from the final analysis.

#### 2.1.3 Ethics and registration

All participants provided written informed consent. The protocol was approved by the Ethics Committee of Tongji Hospital, Tongji Medical College, Huazhong University of Science and Technology (approval number: TJ-IRB202512073) and was registered at ClinicalTrials.gov (NCT07374835).

### 2.2 Intervention protocols

Intervention procedures were delivered for 6 months according to the BMI-specific schedule shown in **Table 1**. Massage was performed three times daily at SP6, SP9, and SP10, with ST36 additionally included for participants with BMI ≥28 kg/m². Duration was 3 min per acupoint for BMI <24 kg/m² and 5–10 min per acupoint for BMI ≥24 kg/m². Dietary guidance was low-salt for BMI 24-27.9 kg/m² and low-GI, high-protein for BMI ≥28 kg/m². The BMI ≥28 kg/m² subgroup also received WeChat-based supervision. Control participants received compression stockings, health education, and aerobic walking as specified in **Table 1**.

**Table 1:** BMI-stratified intervention protocols.

Group	BMI <24 kg/m²	BMI 24-27.9 kg/m²	BMI ≥28 kg/m²
Control group	Compression stockings; health education; aerobic walking (30 min/day, 5 days/week)	Compression stockings; health education; aerobic walking (30 min/day, 5 days/week)	Compression stockings; health education; aerobic walking (30 min/day, 5 days/week)
Intervention group	Meridian massage at SP6, SP9, and SP10 (finger pressure, 3 min/acupoint, 3 times/day); standard health education; follow-up at 1, 3, and 6 months	Meridian massage at SP6, SP9, and SP10 (5–10 min/acupoint, 3 times/day); low-salt dietary guidance; enhanced health education; follow-up at 1, 3, and 6 months	Meridian massage at SP6, SP9, SP10, and ST36 (5–10 min/acupoint, 3 times/day); low-GI, high-protein dietary guidance; compression stockings; WeChat supervision; follow-up at 1, 3, and 6 months

Meridian massage was delivered by qualified rehabilitation therapists who received unified training before the intervention. A written protocol standardized acupoint selection, massage pressure, duration, frequency, and BMI-specific supportive management. Treatment procedures were reviewed during scheduled follow-up visits.

Participants were instructed to record their daily dietary intake and received individualized dietary counselling during follow-up. Intervention adherence was monitored during follow-up but was not quantitatively assessed using a prespecified numerical measure and was therefore not included in the final analysis. The control group received the conventional-care components specified in **Table 1**, and no additional attention-matched intervention was provided.

### 2.3 Outcome measures

The primary outcome was the percentage change in affected-limb volume from baseline to 6 months. Affected-limb circumference was measured at 4-cm intervals using a standardized circumferential measurement protocol, and limb volume was calculated from the serial circumference measurements. The secondary outcome was quality of life, assessed using the validated Chinese version of the LYMQOL. Adverse events, including skin irritation, were recorded throughout the intervention period.

Outcome assessments were performed at baseline and 6 months by uniformly trained researchers who were blinded to group allocation. Formal intra-rater or inter-rater reliability testing was not performed for the circumferential measurements. To minimize measurement variability, all assessors received standardized training and followed the same 4-cm interval measurement protocol.

GCLQ, L-Dex, and tissue hardness were prespecified as additional secondary outcomes in the registered protocol. However, these outcomes were not assessed during the study period and were therefore not included in the final analysis. Accordingly, reliability assessment for tissue hardness was not applicable. Intervention adherence was monitored during follow-up but was not quantitatively assessed using a prespecified metric and was therefore not included in the final analysis.

### 2.4 Statistical analysis

Statistical analyses were performed using SPSS 26.0. Continuous variables are presented as mean ± SD and categorical variables as n (%). The original statistical analysis plan specified independent-samples t tests for between-group comparisons and repeated-measures or longitudinal analyses for outcomes assessed at multiple follow-up time points. In the present manuscript, however, the efficacy analysis was restricted to changes from baseline to 6 months, and intermediate 1- and 3-month outcomes were not included in the final analysis. Therefore, between-group differences in the changes in affected-limb volume and LYMQOL scores from baseline to 6 months were analyzed using independent-samples t tests. Mean between-group differences and corresponding 95% confidence intervals (CIs) were estimated within the same statistical framework. No repeated-measures ANOVA or generalized estimating equation model was applied to the outcomes reported in the present manuscript. This represents a deviation from the original statistical analysis plan and is reported transparently. The comparison of affected-limb volume reduction between the BMI ≥28 kg/m² and BMI 24–27.9 kg/m² subgroups was exploratory. A two-sided P value <0.05 was considered statistically significant.

## 3 Results

### 3.1 Baseline characteristics

Sixty participants were randomized, with 30 assigned to the intervention group and 30 to the control group (**Figure 1**). Baseline demographic and clinical characteristics are presented in **Table 2**. No statistically significant differences were observed between the two groups in age, BMI, BMI category, cancer type, tumor stage, number of lymph nodes dissected, chemotherapy, radiotherapy, or baseline clinical measurements (all P>0.05), indicating generally comparable baseline characteristics between the randomized groups.

**Figure 1:**
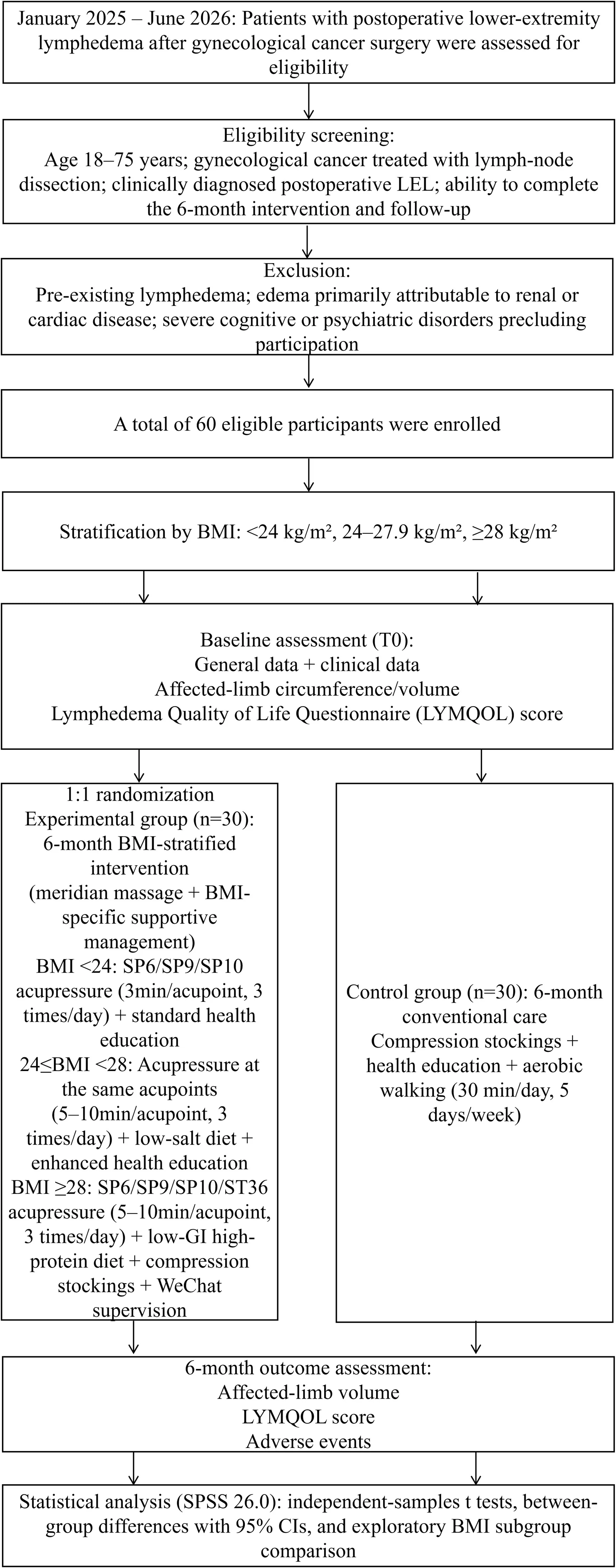
Flow chart.

**Table 2:** Baseline demographic and clinical characteristics of the participants.

Characteristic	Intervention group (n=30)	Control group (n=30)	P-value
Age, years	53.7 ± 8.4	52.9 ± 8.1	0.709
BMI, kg/m²	24.86 ± 3.12	24.71 ± 3.05	0.851
BMI category, n (%)			1
<24 kg/m²	15 (50.0)	15 (50.0)	
24–27.9 kg/m²	10 (33.3)	10 (33.3)	
≥28 kg/m²	5 (16.7)	5 (16.7)	
Cancer type, n (%)			0.793
Cervical cancer	18 (60.0)	17 (56.7)	
Endometrial cancer	9 (30.0)	10 (33.3)	
Ovarian cancer	3 (10.0)	3 (10.0)	
Tumor stage, n (%)			0.787
Early stage	19 (63.3)	20 (66.7)	
Advanced stage	11 (36.7)	10 (33.3)	
Number of lymph nodes dissected	19.4 ± 5.8	18.7 ± 5.5	0.633
Chemotherapy, n (%)	20 (66.7)	21 (70.0)	0.781
Radiotherapy, n (%)	14 (46.7)	13 (43.3)	0.795
Baseline affected-limb volume	8.72 ± 1.36	8.61 ± 1.29	0.749
Baseline LYMQOL score	42.6 ± 5.8	43.1 ± 5.6	0.735

### 3.2 Main results

The comparison of intervention effects at 6 months between the intervention and control groups is presented in **Table 3**. At 6 months, the intervention group showed a significantly greater reduction in affected-limb volume compared with the control group (P<0.01). Similarly, LYMQOL scores improved more in the intervention group than in the control group (P<0.01).

**Table 3:** Comparison of intervention effects at 6 months.

Indicator	Intervention group (n=30)	Control group (n=30)	P-value	Between-group difference (95% CI)
Reduction in affected-limb volume (%)	32.1 ± 6.2	14.3 ± 5.8	<0.01	17.8 (14.7 to 20.9)
LYMQOL score improvement (points)	15.2 ± 3.1	6.8 ± 2.9	<0.01	8.4 (6.8 to 10.0)

#### 3.2.1 Subgroup analysis

An exploratory comparison suggested a larger reduction in affected-limb volume in participants with BMI ≥28 kg/m² than in those with BMI 24–27.9 kg/m² (P = 0.02). This subgroup comparison was not specifically powered, and no adjustment for multiple comparisons was performed. Therefore, this finding should be interpreted as exploratory rather than confirmatory.

### 3.3 Safety

Three of 30 participants (10.0%) in the intervention group experienced transient skin redness that resolved within 24 hours. No serious adverse events were reported.

## 4 Discussion

The present study showed that the BMI-stratified integrated intervention was associated with a greater reduction in affected-limb volume and greater improvement in LYMQOL scores than conventional care after 6 months. Exploratory subgroup analysis also suggested a greater reduction in limb volume among participants with BMI ≥28 kg/m² than among those with BMI 24–27.9 kg/m².

Recent systematic reviews and clinical studies suggest that exercise and combined physical therapy may improve limb volume, symptoms, physical function, and quality of life (13–15). Evidence for acupressure remains limited, although a recent scoping review reported potential benefits in cancer-related lymphedema (16). In the present study, SP6, SP9, SP10, and ST36 were used as standardized stimulation sites within the meridian-lymph conceptual framework. Repeated manual pressure may theoretically facilitate fluid movement through local tissue deformation and changes in interstitial pressure; however, lymphatic flow, vessel contractility, and related biological mechanisms were not directly assessed. Therefore, the observed effects should be interpreted as those of a multicomponent intervention rather than as evidence of an acupoint-specific lymphatic mechanism.

The greater improvement observed with the BMI-stratified intervention may be related to the close association between obesity and impaired lymphatic function. Experimental studies have shown that obesity-related lymphatic dysfunction may involve impaired lymphatic transport, perilymphatic inflammation, and altered lymphatic vessel function, and that some of these abnormalities may improve after weight reduction (17–21). The intervention also incorporated BMI-specific dietary guidance and supportive management. Previous studies have reported that low-GI dietary interventions can reduce body fat and improve metabolic or inflammatory indices in populations with metabolic disorders (22, 23), while structured lifestyle and online support programs may improve participation in weight-management interventions (24). These findings provide a rationale for incorporating dietary and behavioral support into obesity-related lymphedema management. Moreover, because the present intervention combined meridian massage, dietary guidance, compression, health education, exercise, and BMI-specific follow-up, the independent contribution of each component cannot be determined. Although participants with BMI ≥28 kg/m² showed a greater reduction in affected-limb volume than those with BMI 24–27.9 kg/m², this comparison was exploratory, the study was not specifically powered for BMI subgroup analyses, and no adjustment for multiple comparisons was applied. Therefore, this finding should be regarded as hypothesis-generating rather than confirmatory.

This study has several limitations. Although the planned sample size of 60 participants was based on an expected large effect size, the study was not specifically powered for BMI-stratified subgroup analyses, and no adjustment for multiple comparisons was applied; therefore, subgroup findings should be interpreted as exploratory. Participants and therapists could not be blinded, and the control group did not receive attention-matched contact, which may have introduced performance bias, although outcome assessors were blinded. Intervention adherence was not quantitatively assessed, and the multicomponent intervention prevented determination of the independent effects of individual components. In addition, the efficacy analysis was restricted to baseline-to-6-month changes despite longitudinal analyses being specified in the original statistical analysis plan, so intermediate treatment trajectories could not be evaluated. Finally, follow-up was limited to 6 months, and biological mechanisms were not directly assessed. Larger multicenter studies are needed to confirm these findings.

## 5 Conclusion

In this single-center randomized trial with a modest sample size, the 6-month BMI-stratified integrated intervention was associated with greater reductions in affected-limb volume and improvements in LYMQOL compared with conventional care. These findings should be considered preliminary and require confirmation in larger, adequately powered multicenter trials before definitive conclusions regarding clinical efficacy can be drawn.

## Data Availability

The original contributions presented in the study are included in the article/supplementary material. Further inquiries can be directed to the corresponding author.

## References

[1] Cucinella G Di Donna MC Casarin J Schivardi G Multinu F Borsellino L . Lower limb lymphedema after surgical staging for endometrial cancer: current insights and future directions. Taiwan J Obstet Gynecol. (2024) 63:500–5. doi: 10.1016/j.tjog.2024.04.008 39004476

[2] Blum KS Karaman S Proulx ST Ochsenbein AM Luciani P Leroux JC . Chronic high-fat diet impairs collecting lymphatic vessel function in mice. PloS One. (2014) 9:e94713. doi: 10.1371/journal.pone.0094713 24714646 PMC3979858

[3] Hespe GE Kataru RP Savetsky IL García Nores GD Torrisi JS Nitti MD . Exercise training improves obesity-related lymphatic dysfunction. J Physiol. (2016) 594:4267–82. doi: 10.1113/JP271757 26931178 PMC4967732

[4] Executive Committee of the International Society of Lymphology . The diagnosis and treatment of peripheral lymphedema: 2023 consensus document of the International Society of Lymphology. Lymphology. (2023) 56:133–51. doi: 10.2458/lymph.6372 39207406

[5] Forner-Cordero I Muñoz-Langa J DeMiguel-Jimeno JM Rel-Monzó P . Physical therapies in the decongestive treatment of lymphedema: a randomized, non-inferiority controlled study. Clin Rehabil. (2021) 35:1743–56. doi: 10.1177/02692155211032651 34514891

[6] Borman P Koyuncu EG Yaman A Calp E Koç F Sargut R . The comparative efficacy of conventional short-stretch multilayer bandages and Velcro adjustable compression wraps in active treatment phase of patients with lower limb lymphedema. Lymphat Res Biol. (2021) 19:286–94. doi: 10.1089/lrb.2020.0088 33270499

[7] Shinaoka A Koshimune S Suami H Yamada K Kumagishi K Boyages J . Lower-limb lymphatic drainage pathways and lymph nodes: a CT lymphangiography cadaver study. Radiology. (2020) 294:223–9. doi: 10.1148/radiol.2019191169 31746690

[8] Hosaka K Rayner SE von der Weid PY Zhao J Imtiaz MS van Helden DF . Calcitonin gene-related peptide activates different signaling pathways in mesenteric lymphatics of Guinea pigs. Am J Physiol Heart Circ Physiol. (2006) 290:H813–22. doi: 10.1152/ajpheart.00543.2005 16172164

[9] Havas E Parviainen T Vuorela J Toivanen J Nikula T Vihko V . Lymph flow dynamics in exercising human skeletal muscle as detected by scintography. J Physiol. (1997) 504:233–9. doi: 10.1111/j.1469-7793.1997.233bf.x 9350633 PMC1159951

[10] Mukherjee A Nepiyushchikh Z Michalaki E Dixon JB . Lymphatic injury alters the contractility and mechanosensitivity of collecting lymphatics to intermittent pneumatic compression. J Physiol. (2021) 599:2699–721. doi: 10.1113/JP281206 33644884

[11] Lu PH Chen YY Tsai FM Liao YL Huang HF Yu WH . Combined acupoints for the treatment of patients with obesity: an association rule analysis. Evid Based Complement Alternat Med. (2022) 2022:7252213. doi: 10.1155/2022/7252213 35341146 PMC8947926

[12] Lin CH Lin YM Liu CF . Electrical acupoint stimulation changes body composition and the meridian systems in postmenopausal women with obesity. Am J Chin Med. (2010) 38:683–94. doi: 10.1142/S0192415X10008159 20626054

[13] Hsu YY Nguyen TTB Chou YJ Ho CL . Effects of exercise on lower limb lymphedema in gynecologic cancer: a systematic review and meta-analysis. Eur J Oncol Nurs. (2024) 70:102550. doi: 10.1016/j.ejon.2024.102550 38554614

[14] Kara O Mete Civelek G Şahbaz Pirinççi C Tarlabölen R Dalyan M . Effects of aerobic cycling training in patients with gynaecologic cancer-related lower extremity lymphedema: a randomised comparative study. Support Care Cancer. (2025) 33:302. doi: 10.1007/s00520-025-09354-4 40108020

[15] Wu Y Li X Shao P Wang T . Effectiveness of physical therapy for lower limb lymphedema in gynecological cancer survivors: a systematic review of randomized controlled trials. Front Oncol. (2026) 16:1792931. doi: 10.3389/fonc.2026.1792931 41939456 PMC13047141

[16] Shen A Li M Ning H Han G Castelli G Qiang W . The promising application of acupressure for management of cancer-related lymphedema: a scoping review. Asia Pac J Oncol Nurs. (2025) 12:100669. doi: 10.1016/j.apjon.2025.100669 40124657 PMC11930162

[17] Nitti MD Hespe GE Kataru RP García Nores GD Savetsky IL Torrisi JS . Obesity-induced lymphatic dysfunction is reversible with weight loss. J Physiol. (2016) 594:7073–87. doi: 10.1113/JP273061 27619475 PMC5134379

[18] Lu M Yu Z Yang X Yang Z Xu T Yu Z . Electroacupuncture stimulation induced M1/M2 polarization in white adipose tissues by activating the Y1 receptor in obese mice to reduce chronic inflammation. Adipocyte. (2025) 14:2524638. doi: 10.1080/21623945.2025.2524638 40586350 PMC12218457

[19] Lam TF Lyu Z Wu X Wong YP Cao P Wong EY . Electro-acupuncture for central obesity: a patient-assessor blinded, randomized sham-controlled clinical trial. BMC Complement Med Ther. (2024) 24:62. doi: 10.1186/s12906-024-04340-5 38287303 PMC10823622

[20] Torrisi JS Hespe GE Cuzzone DA Savetsky IL Nitti MD Gardenier JC . Inhibition of inflammation and iNOS improves lymphatic function in obesity. Sci Rep. (2016) 6:19817. doi: 10.1038/srep19817 26796537 PMC4726274

[21] Busebee B Ghusn W Cifuentes L Acosta A . Obesity: a review of pathophysiology and classification. Mayo Clin Proc. (2023) 98:1842–57. doi: 10.1016/j.mayocp.2023.05.026 37831039 PMC10843116

[22] Gomes JMG Fabrini SP Alfenas RCG . Low glycemic index diet reduces body fat and attenuates inflammatory and metabolic responses in patients with type 2 diabetes. Arch Endocrinol Metab. (2017) 61:137–44. doi: 10.1590/2359-3997000000206 27598983 PMC10118866

[23] Kaur B Quek Yu Chin R Camps S Henry CJ . The impact of a low glycaemic index (GI) diet on simultaneous measurements of blood glucose and fat oxidation: a whole body calorimetric study. J Clin Transl Endocrinol. (2016) 4:45–52. doi: 10.1016/j.jcte.2016.04.003 29159130 PMC5680450

[24] Wichansawakun S Srikhajonjit W Srichan C Tonglim J . Efficacy of an online exercise program to improve weight management outcomes in individuals with obesity: a randomised trial. Clin Nutr ESPEN. (2025) 66:202–7. doi: 10.1016/j.clnesp.2025.01.039 39880206

